# MicroRNAs, Major Players in B Cells Homeostasis and Function

**DOI:** 10.3389/fimmu.2014.00098

**Published:** 2014-03-11

**Authors:** Richard Danger, Faouzi Braza, Magali Giral, Jean-Paul Soulillou, Sophie Brouard

**Affiliations:** ^1^Institute of Liver Studies, Medical Research Council Centre for Transplantation, King’s College Hospital, King’s College London, London, UK; ^2^Institut National de la Santé et de la Recherche Médicale, U1064, Institut de Transplantation Urologie Néphrologie, Nantes, France; ^3^Faculté de Médecine, Université de Nantes, Nantes, France; ^4^Centre Hospitalier Universitaire, Hôtel Dieu, Nantes, France

**Keywords:** B cell, immunology, immune disorder, microRNA, gene-expression regulation

## Abstract

As a main actor in humoral immunity, B cells participate in various antibody-related disorders. However, a deeper understanding of B-cell differentiation and function is needed in order to decipher their immune-modulatory roles, notably with the recent highlighting of regulatory B cells. microRNAs (miRNAs), key factors in various biological and pathological processes, have been shown to be essential for B-cell homeostasis, and therefore understanding their participation in B-cell biology could help identify biomarkers and contribute toward curing B-cell-related immune disorders. This review aims to report studies casting light on the roles played by miRNAs in B-cell lineage and function and B-cell-related immune pathologies.

## Introduction

**Figure 1:**
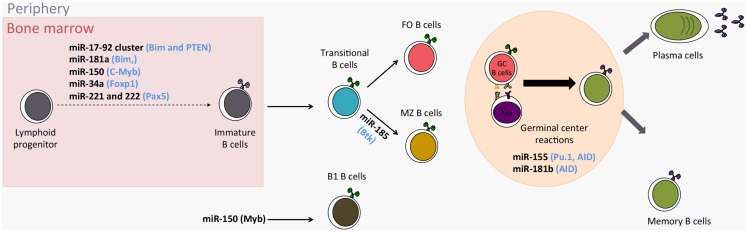
**Role of miRNAs in the development of B cells**. (in Blue: miRNA targets; FO Follicular B cells; MZ Marginal zone B cells).

MicroRNAs (miRNAs) are the most studied class of non-coding RNAs and their gene-expression regulating role is key in various biological and pathological processes. MiRNAs play a role in immune processes such as the development of immune cells, inflammation, and tolerance (1, 2). Evidence that miRNAs are needed for B-cell development is given by mice where B-cell-specific deletion of the endoribonuclease Dicer results in a lack of B cells (3). Furthermore, miRNAs finely tune the differentiation and activation programs of B cells, thus influencing their function. B cells are also central mediators in humoral immunity and play an important role in transplantation, autoimmunity, and reaction to infectious diseases. Consequently, it is important to understand in what circumstances miRNAs can influence B-cell function, and therefore immuno-pathology. In the present review, we describe recent studies shedding light on the roles played by miRNAs in B-cell biology and B-cell-related immune pathologies (major miRNA roles in B cells are reported in Table 1 and Figure 1).

**Table 1:** **Major miRNAs playing a role in B cells**.

miRNA	Targets	Biological effects	Associated disorders
miR-17-92 Cluster	*Bim* (3, 4)	Participate in B-cell proliferation and cell-death control (3, 4)	
	*Pten* (4)		
miR-24	*Bim* and Caspase 9 (5)	Inhibit B-cell development, under the control of PU.1 (5, 6)	
miR-29a	*TCL1*, *MCL10*, and *CDK6* (7)		Up-regulated in indolent B-cell chronic lymphocytic leukemia (CLL) compared to normal B cells (8).
miR-34a	*Foxp1* (9)	Induces block of B-cell development whereas its deletion induces high number of mature B cells (9)	
miR-146a	*Irak1* and *Traf6* (10)	Participate in B-cell development, over-expression causes spontaneous autoimmune disorders in mice (10, 11)	Over-expressed in patients suffering from rheumatoid arthritis and psoriasis (12–14)
	*Fas* (11).		Over-expressed in kidney biopsy and urine from patients with IgA nephropathy (15)
			Induced by EBV and inhibits the expression of interferon related genes (16)
miR-150	*c-Myb* (17).	Its over-expression in B-cell progenitors results in a partial block of B-cell development and a reduction in B1-cell numbers (17).	Under-expressed in peripheral B cells from SLE patients (18)
miR-155	Pu.1 (*Sfpi1* gene) (19)	Reduced generation of high-affinity antibodies against a T-cell-dependent antigen (19, 20)	Over-expressed in peripheral B cells from SLE patients (18)
	*Shp1* (21)		Is induced by EBV, through LMP1 and:
	*Aid* (20, 22)		-targets BMP signaling cascade suggesting an inhibition of the antitumor effects of BMP signaling (23)
			-contributes to the resistance toward Rituximab in inducing cell survival signal (24)
miR-181a	*Bim* (25)	Its over-expression inhibits the pro-apoptotic protein BIM (25) and increases number of B-lineage cells (26)	
miR-181b	*Aid* (27)	Impairs the class-switch recombination (27)	
miR-210		Control of immunoglobin class-switch and under the control of Oct-2 (28)	
miR-221		Implicated in the retention of early B-lineage cells in bone marrow and under the control of PAX5 (29).	

## MicroRNA Characteristics

### Biogenesis

Discovered in *Caenorhabditis elegans* in 1993 by Ambros and Ruvkun’s teams, miRNAs are endogenous small (19–23 nucleotides in length) non-coding RNAs that perform post transcriptional regulation by targeting messenger RNAs (mRNAs) for degradation or translational inhibition (30, 31). Since their first description, miRNAs have been extensively studied. The 20th release (June 2013) of the official miRNA registry, miRbase, contains 2, 578 and 1, 908 mature miRNAs for human and mouse, respectively (32). MiRNA biogenesis has been reported in detail (33). The canonical miRNA biogenesis involves the transcription of long primary transcripts (pri-miRNA) by the RNA polymerase II which allows transcription factor regulation (34). This pri-miRNA is processed by the microprocessor complex, including the endoribonucleases Drosha/Di George syndrome critical region protein 8 (DGCR8). The resulting precursor miRNA (pre-miRNA) is transported into the cytoplasm where it is processed and cleaved by the Dicer RNase III. This process leads to the formation of a short double-stranded RNA containing the miRNA and its complementary sequence. Finally, the mature miRNA is unwrapped and packed in the RNA-induced silencing complex (RISC). This complex is composed of several proteins including the Argonaute proteins (AGO) and allows a stable conservation of the miRNA. This RISC complex guides the miRNA to the target mRNA containing miRNA Recognition Elements (MRE) (35).

### Mechanisms of action

The most widely accepted model for miRNA targeting is based on the seed region, a 6 nucleotide region in the 5′ end of the miRNA, where miRNA/mRNA matching is perfect, whereas an uncomplimentary region, or “bulge” sequence, is present between the miRNA/mRNA hybrid (36). Due to this short recognition sequence, miRNAs are predicted to target hundreds to thousands of genes. This is confirmed in different reports where deletion or over-expression of miRNAs regulate the expression of numerous genes and proteins (37, 38). Consequently, a lot of predictive bioinformatic tools have been developed to identify potential direct miRNA targets [reviewed in Ref. (39)]. However, even the most accurate software gives a high rate of false positives and false negatives and predictions have to be experimentally validated.

The exact mechanisms by which miRNAs repress gene-expression still remain unknown. Recent experiments suggest that miRNAs act as protein transcriptional repressors, preventing ribosome association with mRNAs, leading to mRNA destabilization and degradation (40–42). This would explain the absence of the rapid diminution of mRNA levels after miRNA induction. This would further mean that miRNAs, not requiring translation, could be active in inhibiting mRNA translation more quickly than transcription factors.

Another important property of miRNAs is that they have distinct functions in different cell types, the transcript levels differing depending on the cell, and number of mRNA containing MRE also differing. This is the case for miR-155, which represses the expression of the factor transcription c-MAF and the IFNγ receptor 1 (IFNGR1) in activated naïve CD4 positive cells, whereas it represses the expression of the PU.1 transcription factor and the phosphatidyl inositol 5’-phosphatase SHIP1 in B-lymphocytes [(21, 43, 44); reviewed in Ref. (45)]. Few miRNAs are cell-specific. Some lymphoid miRNAs have been identified, such as miR-150, that have been shown to be expressed in B cells and also in T and NK cells (46). Furthermore, miRNAs have been found expressed in various body fluids including plasma, sera, urine, saliva (47, 48), and their resistance to degradation, either by enzymatic (RNases) or physic (freezing/defreezing) processes make them good biomarkers.

Finally, while the large number of miRNA targets, their possible rapid intervention, and their multifactorial function explain why miRNAs are important in cell biology, the exact mechanisms of miRNA are complex and as yet undiscovered. MiRNAs can directly induce gene-expression (49, 50) despite being mainly described as gene-expression repressors. They can also act in the 5′ untranslated region (UTR), and not only in the 3′ UTR (51). Finally miRNAs can themselves be regulated by long non-coding RNA (52).

## miRNAs and B-Cell Lineage

### Specific miRNAs highlighted in B-cell lineage

Knock-out (KO) experiments have shown that miRNAs are involved in, and even indispensable to, normal hematopoiesis (3, 53) (Figure 1). Indeed, reconstitution of irradiated mice with *Ago2*-deleted bone marrow cells, which induces a reduction in miRNA levels, impairs generation of pre-B cells and subsequently peripheral B cells (53). Specifically in B cells, the *Dicer* ablation at an early stage blocks cells at the pro-B to pre-B-cell transition (3) whereas the *Dicer* ablation at a later stage, in antigen-activated B cells, results in a severe impairment of antibody response with prevention of germinal center (GC) B-cell, long-lived plasma cell, and memory B-cell formation (54). Analysis of up-regulated genes in blocked pro-B cells highlights gene-bearing seed motifs for miRNAs such as miR-142-3p and the miR-17–92 cluster (including miR-17, -19a, -19b, -20a, and -92), suggesting that at least these miRNAs are important for B-cell development (3). Among genes targeted by miRNAs from the miR-17–92 cluster, the authors showed that *Bim* (BCL2-like 11), a pro-apoptotic gene, is increased in *Dicer* KO mice and could be responsible, at least in part, for the massive apoptosis observed at the pre-B stage. In contrast, the over-expression of this miR-17–92 cluster in transgenic mice results in lymphoproliferative and autoimmune phenotypes due to a reduced PTEN (phosphatase and tensin homolog) and BIM protein expression (4). Altogether, these data suggest that the miR-17–92 cluster has a role in the proliferation control of B cells.

Similarly, the over-expression of miR-181a in hematopoietic stem cells induces an increase in the number of B-lineage cells in both tissue-culture and adult mice after re-implantation in bone marrow (26). A deep-sequencing experiment confirms the preferential expression of the miR-181 family in early and transitional B-cell stages (55). Furthermore, the inhibition of the pro-apoptotic gene *BIM* by miR-181a, reducing B-cell apoptosis, could explain the observed increased number of B cells when miR-181a is over-expressed (25). This sequencing study also confirms the expression of miRNAs related to B-cell differentiation, such as miR-146a, miR-150, miR-155, and miR-34a. MiR-150 is indispensable for B-cell development as it controls, in a dose-dependent manner, the expression of *c-Myb* (v-myb avian myeloblastosis viral oncogene homolog, officially called *Myb*), coding for a transcription factor important in multiple steps of lymphocyte development and notably the generation of B1 cells, considered as innate immune cells producing immunoglobulin M and A (IgM and IgA) (17). The ectopic expression of miR-150 in B-cell progenitors results in a partial blockade of B-cell development and a reduction in B1-cell numbers whereas splenic B1 cells are four-times as numerous in miR-150 KO mice (17). Interestingly, in mice with miR-150 ectopic expression, no obvious adverse physiological effect is observed in non-hematopoietic lineage cells, demonstrating that miRNAs display their functions with cell-specificity. Similarly to miR-150, the constitutive expression of miR-34a results in a partial blockade of B-cell development, whereas its deletion induces high number of mature B cells (9). Indeed, miR-34a constitutive expression inhibits the transition of pro-B cells into pre-B cells by targeting the gene coding the transcription factor *Foxp1* (Forkhead box P1). Spierings et al. described high levels of expression of miR-146a in B1 lineage and in to a lesser extent in marginal zone (MZ) B cells (55). Considering that this miRNA has been described as playing a role in a negative feedback loop on NF-kB activity by down-regulating IL-1 receptor associated kinase 1 (*Irak1*) and TNF receptor–associated factor 6 (*Traf6*) upon lipopolysaccharide stimulation in monocyte, miR-146a could be implicated in B1-cell development. The possibility of this role is reinforced by the fact that miR-146a controls *Irak1* and *Traf6* in splenic B cells (10) and furthermore by the association of increased miR-146a expression in *c-Myc* related lymphoma models (56) and in splenic MZ lymphoma (57). Thus, it is clear that miRNAs play key roles in B-cell development.

As for other RNAs, the expression of miRNAs is under the control of transcription factors, which are definitely involved in B-cell development. Starting from the fact that PAX5 (paired box 5) participate in B-cell fate, Knoll et al. have shown the down-regulation of miR-221 and miR-222 during B-lymphocyte development, and the involvement of miR-221 in the retention of early B-lineage cells in bone marrow (29). A similar procedure has been shown with the transcription factor PU.1 {encoded by the *Sfpi1* gene [SPI1 spleen focus forming virus (SFFV) proviral integration oncogene]}, because high ectopic expression of *Pu.1* in multipotent progenitors promotes myeloid cell development at the expense of B-cell development (6). Using a *Pu.1^−^*^/^*^−^*myeloblast cell line and model of bone marrow transplantation, the authors identified miR-24 as a transcriptional target of PU.1 able to inhibit B-cell development *in vivo* as well as *in vitro* (6). In contrast, the same group report that miR-24 enhances cell survival in both the myeloid and pre-B-cell lines, inhibiting pro-apoptotic molecules such as BIM and Caspase 9 (5). It remains unclear why this miRNA inhibits B lymphopoiesis or enhances lymphocyte survival but is likely due to a change in the cellular environment.

Regarding the other roles of miRNAs in B cells, it is clear that they partly control proliferation and apoptosis in B cells and numerous miRNAs have been highlighted in lymphoma. B lymphoma-related miRNAs were reviewed recently (58). Their study highlighted the role of miRNAs in normal B cells. For example, miR-155, which was initially described within the non-coding B-cell integration cluster (*BIC*) gene, is over-expressed in various lymphoma, and has also been shown to be a major miRNA involved in B-cell maturation. This is also the case for miR-29a, highly expressed in B cells and up-regulated in indolent B-cell chronic lymphocytic leukemia (CLL) compared to normal B cells (8). Its B-cell-specific over-expression induces a CLL-like disease in mice with an expanded CD5^+^CD19^+^ B-cell population suggesting that miR-29a acts as an oncomiR (8). However, this miRNA is expressed less in aggressive CLL compared to indolent CLL, and it is speculated that a reduction of control of miR-29a targets, including several oncogenes such as T-cell leukemia/lymphoma 1A (*TCL1*), myeloid cell leukemia sequence 1 (*MCL10*) and cyclin-dependent kinase 6 (*CDK6*) participate to the aggressive CLL phenotype (7). Thus, miR-29a could act either as an oncogene or a tumor suppressor, demonstrating that miRNAs can play different roles depending on the cellular context. Finally, the role of miR-29a in normal B cells has not yet been described, but considering the enrichment of B-cell signaling pathways among its targets, it is also likely to have an important function in normal B cells [reviewed in Ref. (59)].

### Enriched miRNAs in B-cell lineage

Overall, most miRNAs are ubiquitously expressed, only some of them being preferentially expressed, in restricted cell types (60, 61); this is the case for miR-122 in liver and miR-1 in muscle. Global profiling studies focusing on hematopoietic cell lineage and particularly in B cells also foreground enriched miRNAs such as miR-16, miR-30c, miR-34a, miR-142-3 and-5p, miR-150, miR-155, miR-181, and miR-223 (46, 55, 62–65). These profiling studies are very useful tools in a first attempt to appreciate the role of a particular miRNA in conjunction with cells or tissues expressing this miRNA. This is the case for the deep-sequencing study by Spierings et al. which shows the expression of 232 known miRNAs in 10 developmental stages (55). Altogether, these studies demonstrate that miRNAs with distinct expression in B cells have to be investigated for the complete understanding of B-cell biology.

## Implication of miRNAs in Peripheral B-Cell Development

### Roles of miRNAs in FO MZ fate decision

Primary antibody diversification takes places during B-cell differentiation in the bone marrow through somatic DNA rearrangement of Ig by V(D)J recombination. This process leads to a high diversity of B-cell antigen receptors that can recognize self-antigens and potentially induce autoimmunity. Control of B-cell auto-reactivity is guaranteed by intrinsic tolerance mechanisms (66, 67). Two checkpoints ensure B-cell tolerance in bone marrow and the periphery, where strongly self-reactive B cells might undergo receptor editing or clonal deletion (66, 67). In mice, peripheral immature IgM^+^ B cells start to express IgD and terminate their differentiation into follicular (FO) or MZ B cells (11, 68). FO B cells are currently associated with a T dependent-response whereas MZ B cells, located in the spleen marginal sinus, can mount independent humoral responses. The differentiation of immature B cells into FO or MZ B cells depends on BCR intensity signals and cleavage of Notch2. For their differentiation MZ B cells need weak BCR signals, allowing the activation of Notch2 pathways, whereas FO B cells are generated after strong, tonic BCR interactions associated with BAFF survival signals (11). MiRNA are involved in the FO vs. MZ fate decision (69). Mice with conditional KO of *Dicer* in B cells exhibit a total switch of their B-cell subsets, with a higher proportion of immature and MZ B cells and a strong alteration of the FO B cells (69). This alteration is specific to *Dicer* deletion and not due to any compensatory homeostatic mechanisms. Mixed chimeric mice, with 50% wild-type bone marrow and 50% conditional *Dicer* KO bone marrow, exhibit an impairment of FO B-cell generation and an increase in the MZ compartment only in the KO part. This confirms that the augmentation of MZ B cells in *Dicer*-deficient mice is not a homeostatic response but instead reflects an altered process in B-cell fate induced by the absence of miRNAs (69). Remarkably, FO B cells have a higher expression of *Dicer* than MZ B cells, suggesting a central role for miRNAs in this population. Indeed, among the 177 measured, 31 differentially expressed miRNAs have been highlighted in FO and MZ B cells. Among them, miR-185 was identified as central for the differentiation into the FO compartment (69). MiR-185 targets burton tyrosine kinase (*BTK*), which transduces signals downstream of BCR by phosphorylating Erk pathway. Consequently, by targeting *BTK*, miR-185 modulates BCR signals and its activation threshold. Thus, in physiological conditions, miR-185 dampens BCR signals, confirming that immature B cells needs strong BCR activation to differentiate into FO B cells (70). In *Dicer*-deficient mice, BCR signals are not diminished and so immature B cells preferentially differentiate into the MZ compartment. In addition, *Dicer* KO mice exhibit autoimmune features with a skewed antibody repertoire enriched in self-reactive specificities that lead to the development of autoimmune diseases (69). Concentration of IgG against dsDNA, ssDNA, and cardiolipin autoantigens are increased in *Dicer* KO mice, suggesting a passive biased selection of the BCR repertoire, probably due to alterations in BCR signals. More interestingly, these autoimmune features are only found in older female mice, mimicking thus the etiology of autoimmune diseases in human where older women are more susceptible to developing such pathologies (71).

### Roles of miRNAs in the control of memory and humoral responses

Memory and plasma cells are generated during the primary immune response against foreign antigens. This process is initiated when naïve FO and MZ B cells expressing surface Ig bind the antigen in secondary lymphoid organs, receive or do not receive signals from helper T cells, and proliferate. This proliferation produces short-lived plasmablasts and GC cells. A secondary diversification process occurs in the GC where B cells switch their Ig constant region from IgM to IgG, IgA, or IgE and generate somatic mutations in their variable regions. B cells expressing high affinity Ig survive and emerge from the GC reaction and differentiate into plasma cells. Recently, a number of studies have identified the involvement of miRNAs in the GC reaction and B-cell memory responses. Mice lacking miR-155 exhibit substantial immune defects with reductions in GC B cells and dampened B-cell memory responses accompanied by an alteration in the function of T lymphocytes and dendritic cells (43, 72). miR-155 deficiency in B cells leads to a reduced generation of high-affinity antibodies against a T-cell-dependent antigen (19). The authors have identified the transcription factor PU.1 among targets of this miRNA. Furthermore, they have shown that *Pu.1* over-expression in B cells results in reduced numbers of IgG1-switched cells, reinforcing the evidence of this factor in B-cell maturation. In addition, the gene coding for the activation-induced cytidine deaminase (*Aid* gene) regulating the class-switch recombination and somatic hypermutation, has also been shown to be a miR-155 target (20, 22). *Aid* is also a target gene for miR-181b, impairing the class-switch recombination, and its expression is down-regulated upon B-cell activation, allowing efficient antibody maturation (27). Although the majority of miRNAs are down-regulated upon B-cell activation, miR-210 has been shown to be up-regulated in these circumstances (28). In models of KO and transgenic mice, miR-210, itself under the control of the transcription factor OCT-2, is involved in the control of immunoglobin class-switch preventing autoimmunity (28) and mice deficient in miR-210 spontaneously produce high levels of autoantibodies.

Collectively these data demonstrate that miRNAs have a key role in the differentiation of peripheral mature B cells and in humoral responses. In addition, the data suggest that deregulation of miRNA expression can alter B-cell homeostasis and break tolerance by favoring the generation of autoantibodies. Regarding the significance of miRNAs in B-cell biology, we can also assume that miRNAs are involved in other essential functions of B-cells. However, to our knowledge, no miRNA has yet been described as playing a direct role in B cells functions such as antigen presentation to T cells, cytokine secretion or regulatory functions (73).

## miRNA Dysregulation in B-Cell-Related Disorders

### B-cell-related miRNAs in autoimmune disorders

B-cells are involved in autoimmune diseases due to their primary function of antibody production. That miRNA plays a role in the establishment of autoimmunity has been strongly suggested in rodents (69), but few studies have been performed specifically on human B cells. Stagakis et al. found seven miRNAs with differential expression in peripheral B cells in a small group of 5 patients with SLE, compared to three healthy controls (18). Three were under-expressed in SLE (miR-150, miR-16, miR-15a) and the four others were up-regulated (miR-155, miR-25, miR-21, miR-106b). Interestingly, miR-21, a pleiotropic miRNA described as controlling major cell functions, had also been shown to be over-expressed in splenic B cells from two mice models of SLE, the MRL/lpr and the B6.Sle123 mouse strains, suggesting it has a role in this pathology (74, 75). Among others, miR-15a modulation has also been described in another SLE model, B/W mice enhanced by IFNλ, with a significant correlation between miR-15a expression and autoantibody production in SLE prone-B/W mice (76). Surprisingly, miR-15a expression is essentially found in the regulatory B-cell subset under steady state and its expression progressively increases in other B-cell subsets along the course of the disease. However, in this study, miR-15a is over-expressed in splenic B cells, although it has previously been described as down-expressed in human blood B cells (18). Further investigation is required to decipher whether this discrepancy is due to differences in the origins of the analyzed B cells (peripheral blood vs. splenic B cells) or to differences between the mice model and human SLE. Even if miRNA sequences are well conserved between species, their expression profiles could be different from one species to another. For example, among 12 analyzed miRNAs which were expressed both in human and mouse, only 6 had similar expression profiles in their lymphocyte subsets (mainly CD4^+^ cells) (77). Using a hypothesis-driven approach, miR-30a was shown to directly target Lyn, a member of the Src family preferentially expressed in B cells (78). Gene and protein levels of Lyn are lower in SLE patients and negatively correlate with the expression of miR-30a in blood CD19^+^ purified cells (78). Unlike other members of the miR-30 family, only miR-30a exhibits regulatory capacity upon B-cell proliferation and antibody production in the two B-cell lines studied (Daudi and Raji). Interestingly, miR-142-3p and -5p, which are down-regulated in CD4^+^ T cells from SLE patients, have an indirect effect on IgG production; the over-expression of these miRNAs in SLE CD4^+^ T-cells induces a decrease in IgG production (79). In multiple sclerosis (MS), two studies from the same group show two sets of modulated miRNAs in isolated B cells from relapsing-remitting MS patients compared to healthy controls (80, 81). Despite no overlapping miRNAs, five differential miRNAs from the second study have been validated in a second set of samples and could thus be investigated to decipher B-cell deregulation in MS (miR-106b, miR-19b, miR-181a, miR-25, and miR-93) (81).

The over-expression of miR-146a has been reported in synovial tissue from patients with rheumatoid arthritis. *In situ* hybridization analysis reveals that CD79A^+^ B cells express high amounts of miR-146a in these synovial tissues (13). Similarly, miR-146a is over-expressed in kidney biopsy and urine from patients with IgA nephropathy, although its specific expression has not been proven (15). The involvement of miR-146a in immune disorders is further suggested by the generation of transgenic mice over-expressing miR-146a with immune disorders including enlarged spleens and lymph nodes, increased frequency and numbers of T and B cells, accumulation of GC B cells, and an increase in Ig serum levels (11). MiR-146a mediates its effect by repressing the expression of Fas in B cells, a molecule essentially expressed on GCB cells and which promotes their apoptosis during GC reaction. Thus over-expression of this miRNA enhances homeostatic B-lymphocyte proliferation leading to the development of autoimmune lymphoproliferative syndrome. In concordance with these observations, higher levels of miR-146a have been found in patients suffering from rheumatoid arthritis and psoriasis (12–14).

### B-cell-related miRNAs in solid organ transplantation

An increasing number of articles dealing with miRNAs and solid organ transplantation, including kidney, liver, and lung, suggest their significant role in organ acceptance or rejection and their usefulness as biomarkers (82, 83). Regarding B cells, several miRNAs have been identified in biopsy or peripheral blood mononuclear cells from patients with antibody-mediated rejection (AMR) in renal transplantation (84–86). The link between these miRNAs and B cells is probable, but because B cells can exercise their function at a distance from the graft and because other factors participate in the AMR process, further investigation is required to demonstrate that these miRNAs are really involved. To our knowledge, we were the first to report miRNA dysregulation specifically in B cells after transplantation. We reported the over-expression of miR-142-3p in blood B cells from patients with operational tolerance, a specific situation where transplant patients maintain a well-functioning graft having stopped their immunosuppressive treatment (87). The over-expression of miR-142-3p in the Raji B-cell line induces the modulation of genes previously described as associated with renal tolerance, suggesting that it may contribute to the maintenance of tolerance in B cells. These observations were reinforced by the observation that miR-142-3p may play a regulatory role in T lymphocytes by controlling leukocyte activation (79). In addition, miR-155 has been shown to contribute to resistance to Rituximab in inducing cell survival signals through AKT and myeloid cell leukemia sequence 1 (MCL1) since its inhibition resulted in a significant decrease in the survival of EBV-positive cells treated with Rituximab (24). These results indicate that the inhibition of miR-155 could be a valuable approach for treating EBV-induced PTLD.

Of course, other phenomena not directly related to B cells could occur during transplantation, including ischemia and reperfusion, cellular rejection, and recurrence of the initial disease, and miRNAs could be associated with these. In renal transplantation, miR-142-5p, shown to be present during chronic AMR, has been previously indicated as over-expressed in biopsies from patients with cellular acute rejection (85, 88). Similarly, miR-142-3p, related to B cells in renal operational tolerance, has been found to be associated with interstitial fibrosis and tubular atrophy in urine (87, 89).

### B-cell-related miRNA usefulness in therapy

Since miRNAs can be detected and measured in various body fluids, they may represent ideal non-invasive biomarkers. Among recent, numerous examples, miR-210 has been proposed as an urinary biomarker of acute rejection in renal transplantation (48), miR-142-5p to diagnose chronic AMR in renal allograft (85), and miR-155 to predict patients with CLL who fail to achieve a complete response in plasma samples collected before treatment initiation (90).

As a result, the use of miRNA inhibitors, otherwise known as antagomirs, may be promising as therapeutic tools. One particular example is the use of miRNA inhibitor against miR-122, a liver specific microRNA required by the hepatitis C virus (HCV) for replication. The use of this miR-122 inhibitor, “miravirsen,” induced a decrease in HCV RNA levels in a dose-dependent manner in a clinical phase II study (91). Other inhibitors of miRNAs have been proposed for various diseases and it can be assumed that B-cell targeting ones will be designed in the future, for example to impair their production of antibodies. However, the targeted miRNAs generally have several functions in several different cell types and while their inhibition could provide powerful remedies, they could also have wide-reaching side-effects and caution is mandatory.

Finally, it has recently been proposed to use B cells as producers and delivers of therapeutic miRNAs in CD8^+^ T cells (92). After *in vitro* transfection of B cells with a plasmid coding for miR-155, these B cells delivered miR-155 in CD8^+^ T cells during antigen cross-priming only (92). The authors suggest this cell-based strategy could be used in inflammation and autoimmune diseases.

## Conclusion

Recent studies have clearly demonstrated that miRNAs are involved in B-cell development and function. With the current interest in miRNAs, as well as the renewed emphasis on B-cell function, notably triggered by the recent discovery of regulatory B cells, it seems clear that further discoveries will be made in the near future. Ideally, theses insights would allow the use of miRNAs as disease biomarkers, but may also allow modulation of miRNA expression as master gene modulators to cure B-cell-related immune disorders.

## Conflict of Interest Statement

The authors declare that the research was conducted in the absence of any commercial or financial relationships that could be construed as a potential conflict of interest.
